# Sequential Analysis of a Panel of Biomarkers and Pathologic Findings in a Resuscitated Rat Model of Sepsis and Recovery

**DOI:** 10.1097/CCM.0000000000002381

**Published:** 2017-07-14

**Authors:** Nishkantha Arulkumaran, Marije L. Sixma, Elisa Jentho, Elias Ceravola, Paul S. Bass, John A. Kellum, Robert J. Unwin, Fred W. K. Tam, Mervyn Singer

**Affiliations:** 1 Bloomsbury Institute of Intensive Care Medicine, Division of Medicine, University College London, London, United Kingdom.; 2 Department of Nephrology, Division of Medicine, University College London, London, United Kingdom.; 3 Hammersmith Hospital, Imperial College Renal and Transplant Centre, London, United Kingdom.; 4 Department of cellular pathology, Royal Free Hospital, London, United Kingdom.; 5 Center for Critical Care Nephrology, Department of Critical Care Medicine, University of Pittsburgh, Pittsburgh, PA.

**Keywords:** acute kidney injury, animal model, biomarkers, sepsis

## Abstract

**Objectives:**

To characterize the temporal pattern of a panel of blood and urinary biomarkers in an animal model of fecal peritonitis and recovery.

**Design:**

Prospective observational animal study.

**Setting:**

University research laboratory.

**Subjects:**

Male Wistar rats.

**Interventions:**

A fluid-resuscitated, long-term (3 d) rat model of sepsis (fecal peritonitis) and recovery was used to understand the temporal association of sepsis biomarkers in relation to systemic hemodynamics, inflammation, and renal function. At predefined time points (3, 6, 12, 24, 48, 72 hr), animals (≥ 6 per group) underwent echocardiography, blood and urine sampling, and had kidneys taken for histological analysis. Comparison was made against sham-operated controls and naïve animals.

**Measurements and Main Results:**

The systemic proinflammatory response was maximal at 6 hours, corresponding with the nadir of stroke volume. Serum creatinine peaked late (24 hr), when clinical recovery was imminent. Histological evidence of tubular injury and cell death was minimal. After a recovery period, all biomarkers returned to levels approaching those observed in sham animals. Apart from urine clusterin and interleukin-18, all other urinary biomarkers were elevated at earlier time points compared with serum creatinine. Urine neutrophil gelatinase-associated lipocalin was the most sensitive marker among those studied, rising from 3 hours. While serum creatinine fell at 12 hours, serum cystatin C increased, suggestive of decreased creatinine production.

**Conclusions:**

Novel information is reported on the temporal profile of a panel of renal biomarkers in sepsis in the context of systemic and renal inflammation and recovery. Insight into the pathophysiology of acute kidney injury is gleaned from the temporal change markers of renal injury (urine neutrophil gelatinase-associated lipocalin, kidney injury molecule-1, calbindin), followed by a marker of cell cycle arrest (urine insulin-like growth factor-binding protein 7) and, finally, by functional markers of filtration (serum creatinine and cystatin C). These clinically relevant findings should have significant influence on future clinical testing.

## Abstract 2

Supplemental Digital Content is available in the text.

Sepsis is the underlying cause in half the cases of acute kidney injury (AKI) (1). Up to 5% of total ICU admissions require renal replacement therapy for AKI and a third of them die (1, 2). Despite the clinical impact of septic AKI management is limited to supportive care. The current lack of a sensitive marker of early parenchymal kidney injury reduces the window of opportunity for early effective intervention to prevent renal dysfunction and failure. Numerous blood and urine markers of renal injury/dysfunction are being promoted but, at present, remain poorly characterized. Few studies have investigated the utility of biomarkers in predicting recovery from AKI (3). A complementary panel of markers will likely enhance interpretation of this dynamic process, although studies in septic patients are confounded by an inability to precisely time the onset of sepsis. Thus, the temporal relationship of these biomarkers to the onset, progression, and recovery of renal dysfunction and injury is unknown. Serial measurements of a panel of renal biomarkers in a well-characterized animal model with a defined onset of polymicrobial sepsis followed by recovery will provide invaluable information translatable to the patient.

Animal models of sepsis offer the advantage of knowing precisely when “time zero” occurs and also allow control of volume status and other conditions in a relatively homogenous population. However, to be representative of the human condition, they must simulate many of the physiologic and pathologic aspects of sepsis, including a proper infectious insult, an adequate duration of study, and fluid resuscitation to avoid the consequences of untreated hypovolemia leading to organ hypoperfusion. We used a clinically relevant and well-characterized model of sepsis and recovery to define parallel changes in global hemodynamics, biochemistry, renal histology, serum cytokines, and a panel of renal biomarkers (4, 5).

## MATERIALS AND METHODS

### In Vivo Experiments

Male Wistar rats (Charles River, Margate, Kent, United Kingdom) weighing 300–375 g were used. Experiments were performed under a Home Office Project License (PPL 70/7029) and local University College London Ethics Committee approval. The rats were housed in cages of six on a 12-12–hour light-dark cycle. Six time points were selected to represent early (3, 6, 12 hr), established (24 hr), and recovery (48 and 72 hr) phases of sepsis.

All invasive and imaging techniques were performed under a brief period of isoflurane anesthesia with the animal breathing spontaneously, as described previously (4, 5). Following tunneled internal jugular line placement, rats were placed in individual cages mounted on the tether/swivel system to secure the IV catheter and allow unimpeded movement with free access to food and water. The reasons for tunneled central venous catheter (CVC) insertion are two-fold; first, to prevent the line from being pulled out by the animal and thus enable ongoing fluid resuscitation and second, to reduce the risk of CVC-related infection. At 24-hour postinstrumentation, sepsis was induced by intraperitoneal injection of fecal slurry (see **Appendix** for more detail). This was not performed in sham animals to prevent inadvertent bowel perforation.

Antibiotics were not administered to avoid any confounding drug-induced nephrotoxicity. The optimal volume and rate of fluid administration (with 1:1 mix of 5% glucose and Hartmann’s solution) to maintain intravascular volume based on echo variables have been previously determined (4). Sham animals received a similar fluid regimen.

Echocardiography was performed prior to each fluid bolus and at the terminal time point as previously described (4, 5). At the terminal time point, a carotid arterial catheter was inserted under isoflurane anesthesia with the animals breathing spontaneously. Blood gas analysis was performed using 0.2 mL arterial blood taken into heparinized capillary tubes (ABL-70 analyzer, Radiometer, Copenhagen, Denmark).

A laparotomy incision was made and a 22-gauge needle used to aspirate urine via bladder puncture. The left kidney was isolated and the upper pole placed into formalin and the rest snap-frozen in liquid nitrogen. Cardiac puncture was then performed to obtain blood which was placed in a heparinized tube and centrifuged at 6,500 rpm for 10 minutes. The serum was siphoned off, aliquots taken, snap-frozen in liquid nitrogen, and stored at –80°C.

### Serum and Tissue Sample Measurements

DuoSet enzyme-linked immunosorbent assay (ELISA) kits (R&D Systems, Minneapolis, MN; BD Biosciences, Oxford, Oxon, United Kingdom) were used to assess serum cytokine levels according to the manufacturers’ instructions. Absorbance was read at 450 nm using a spectrophotometric ELISA plate reader (Anthos HTII; Anthos Labtec, Salzburg, Austria).

MILLIPLEX_MAP_ multianalyte panels (Merck Millipore, Watford, Herts, United Kingdom) were used for simultaneous detection and quantification of eight biomarkers and cytokines/chemokine in rat urine, including neutrophil gelatinase-associated lipocalin (NGAL), cystatin C, interleukin (IL)-18, monocyte chemotactic protein (MCP)-1, clusterin, calbindin, osteopontin, and kidney injury molecule (KIM)-1. The same technique was used to determine serum levels of IL-18. Assays were performed according to the manufacturer’s protocols. The plate was read on a Bio-Plex 200 multiplex system (Bio-Rad, Hemel Hempstead, Herts, United Kingdom). Urine tissue inhibitor of metalloproteinases-2 (TIMP-2) and insulin-like growth factor-binding protein 7 (IGFBP7) were analyzed by ELISA as described previously (6). Renal function (serum creatinine) was analyzed using the Jaffe assay by the Clinical Pathology laboratory at the Royal Free Hospital, London, United Kingdom. Where urine biomarkers were analyzed, matched creatinine values were used.

### Immunohistochemistry

Kidneys were fixed for 24–72 hours in formalin, transferred to 70% ethanol, and embedded in paraffin. Sections were then cut into 5 µm slices and mounted onto glass slides. For all histological analyses, sections were examined using an Olympus B×4 microscope (Olympus Optical, London, United Kingdom) at ×20 magnification. For assessment of tubular injury (tubular dilatation, brush border loss, and tubular cast formation), sections were stained with Periodic acid-Schiff. Ten random fields of view of the cortex were analyzed for each section at ×20 magnification under a light microscope. Apoptosis was identified by DNA fragments in situ using the terminal deoxyribonucleotidyl transferase (TdT)-mediated biotin-16-2-deoxyuridine 5'-triphosphate (dUTP) nick-end labeling (terminal deoxynucleotidyl transferase dUTP nick-end labeling [TUNEL] assay) using the TACS TdT In Situ Apoptosis Detection Kit (R&D Systems, Abingdon, Oxford, United Kingdom). The total number of apoptotic bodies per ×20 field was counted manually and an average taken for each group. A total of three slides for the septic groups (with at least one from the poor prognosis subgroup) and two from the naïve groups from time points 6, 12, 24, 48, and 72 hours were selected.

### Statistics

Analyses were performed using SPSS (IBM SPSS Statistics, Version 22.0; IBM Corp, Armonk, NY), and graphs drawn using Graphpad Prism Version 5.0d (GraphPad Software, La Jolla, CA). Continuous variables are presented as median (interquartile range). Differences in continuous variables between groups were compared using Mann-Whitney *U* test. Pearson’s correlation was performed to assess the degree of correlation between serum and urine levels of biomarkers (cystatin C, NGAL, IL-18, and MCP-1). A *p* value of less than 0.05 was taken as statistically significant.

## RESULTS

Baseline measurements (weight, temperature, heart rate [HR], stroke volume [SV], cardiac output [CO]) were similar between groups. In total, 19 animals died (20%, as per the severity of the model). All deaths occurred after the 12-hour time point and before the 48-hour time point. These animals were not used for biomarker/biochemistry analyses. The sampling time (involving kill of the animals) was randomized. All animals intended for sampling at early time points survived, whereas some of the animals intended for sampling at a late time point succumbed prior to reaching this time point.

### Physiologic Markers

**Figure 1.**
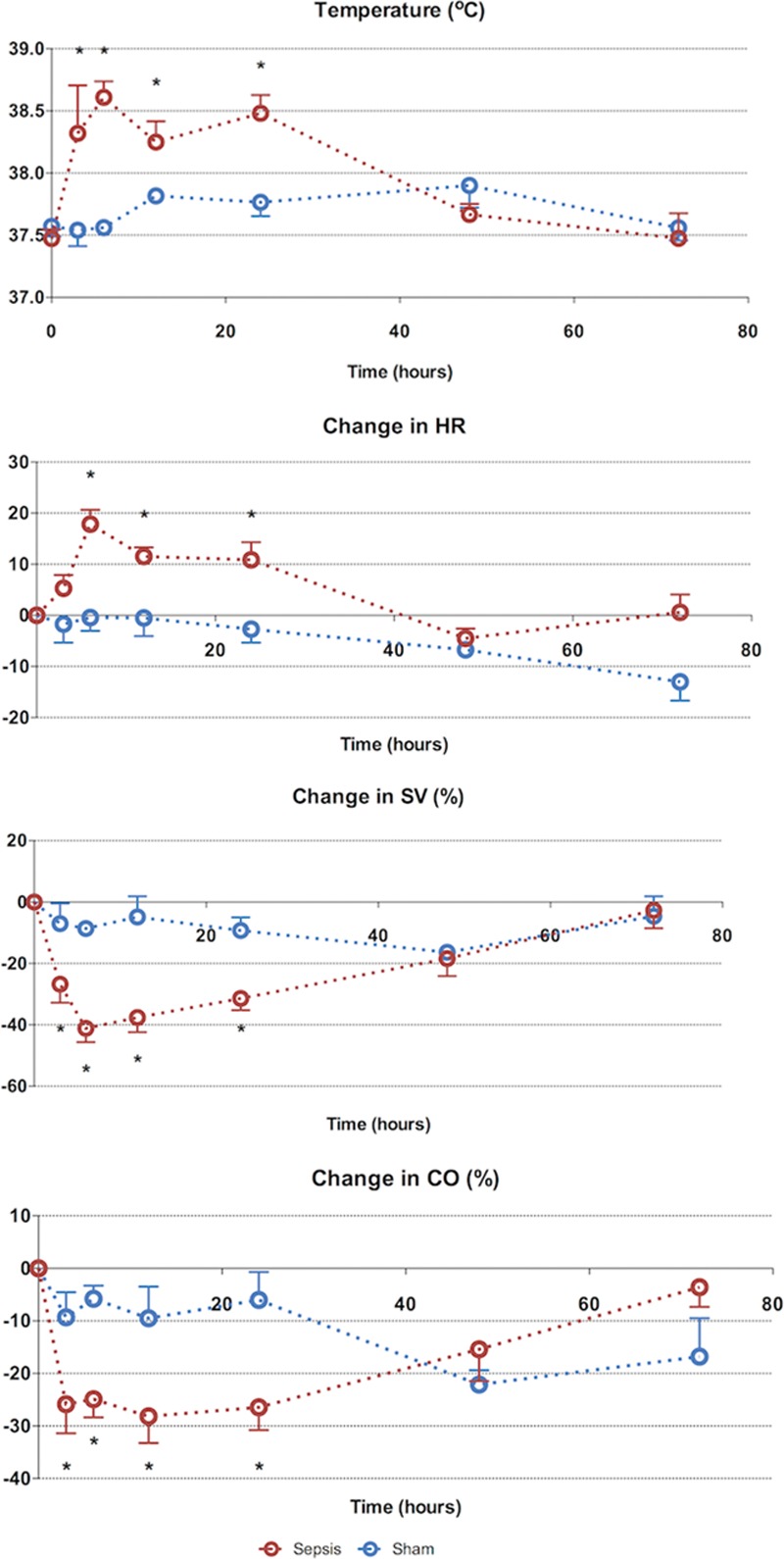
Seventy-two–hr characterization of sepsis and recovery phases—systemic variables. Septic animals develop a tachycardia, fall in stroke volume (SV) and fever early (3–6 hr), which resolves from 24 hr to reach baseline values at 72 hr. *Points* and *whiskers* represent median and interquartile range, respectively. *p < 0.05 sham vs sepsis; (*)*p* = 0.05–0.06 sham vs sepsis. CO = cardiac output, HR = heart rate.

At 3-hour postinduction of sepsis, there was a significant fall in stroke volume and cardiac output (**Fig. 1**). Septic animals mounted a significant tachycardia by 6 hours, which persisted at 24 hours. This paralleled the increase in core body temperature. By 48 hours, SV, CO, HR, and core temperature normalized among septic animals, and these remained stable until 72 hours.

### Biochemical Markers

**Figure 2.**
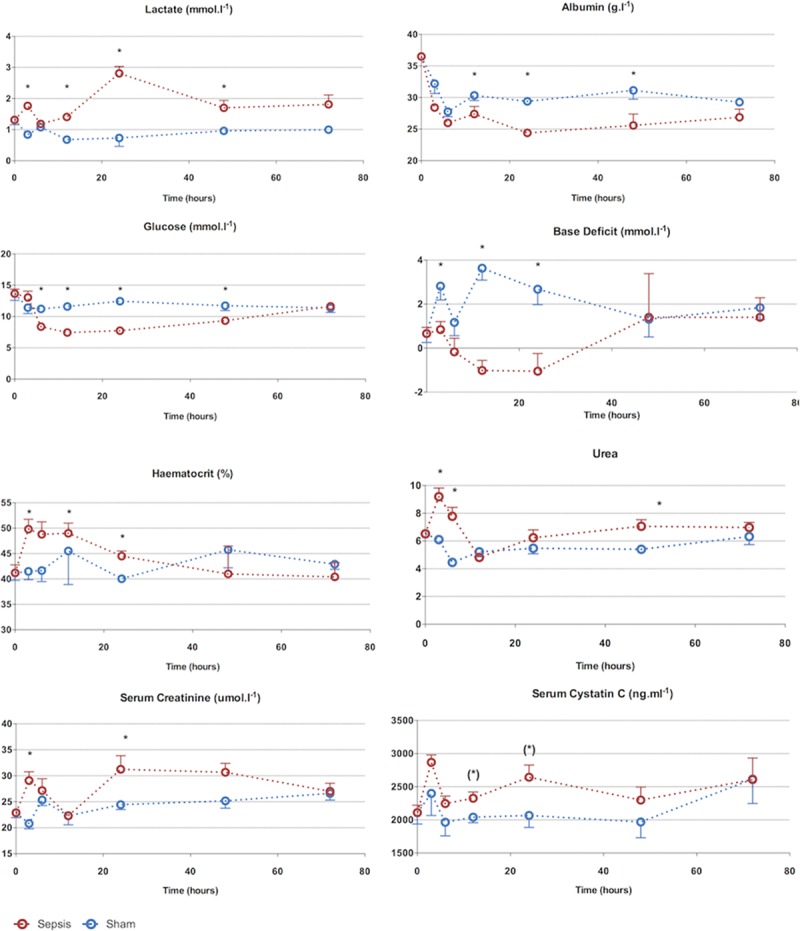
Seventy-two–hr characterization of sepsis and recovery phases—biochemistry. Biochemical changes occur from 3 hr. Apart from serum urea, changes are maximal at 24 hr followed by recovery to baseline values at 72 hr. The early rise in urea, creatinine, and lactate is corrected after fluid resuscitation, demonstrating early hemoconcentration. *Points* and *whiskers* represent median and interquartile range, respectively. *p < 0.05 sham vs sepsis; (*)*p* = 0.05–0.06 sham vs sepsis.

There was an early peak (3 hr) in serum urea and creatinine in septic animals (**Fig. 2**). Alongside the significant rise in hematocrit at 6 hours, this suggests intravascular volume depletion, despite aggressive fluid loading. Once fluid resuscitation commenced (from 2 hr), there was a progressive fall in serum urea and creatinine that normalized by 6–12 hours. By 24 hours, serum urea and creatinine were significantly elevated. The peak serum creatinine level (30 µmol/L) was 1.5-fold above that of baseline. The increases in arterial lactate levels were phasic, with a rise at 3 hours, normalization at 6 hours, a further peak at 24 hours and then a subsequent fall. The arterial base excess fell in the septic animals but normalized by 48 hours. The rise in serum cystatin C approached statistical significance by 12 hours and remained elevated at 24 hours. Compared with sham animals, serum albumin and glucose fell in the septic animals, reaching a nadir at 24 hours. This change was more pronounced in the septic rats compared with sham-operated rats. Recovery occurred by 72 hours.

### Serum Cytokines

**Figure 3.**
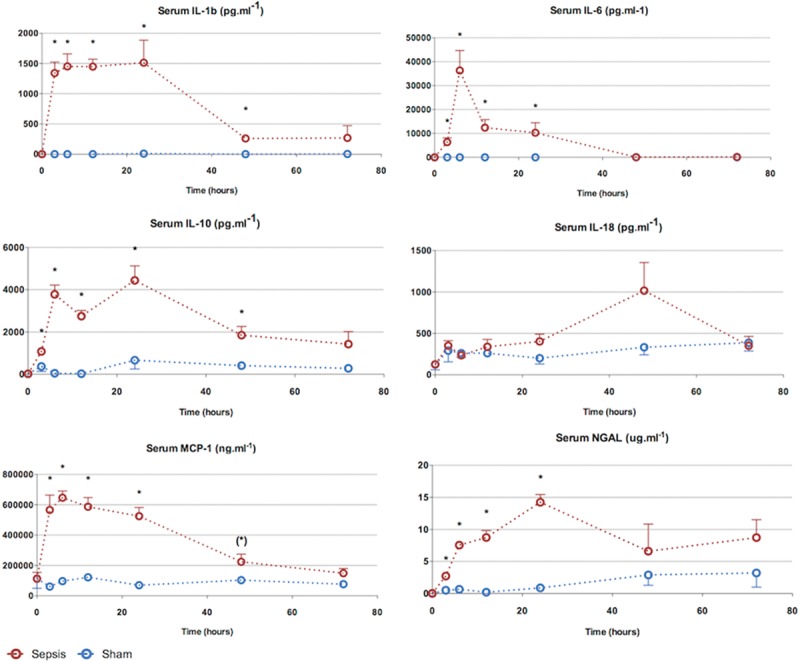
Characterization of sepsis and recovery phases—serum cytokines. The proinflammatory cytokine kinetic pattern is variable. Interleukin (IL)-1β and IL-6 rise early (3 hr) and remain elevated until 24 hr. The anti-inflammatory cytokine IL-10 begins to rise by 3 hr but remains elevated through to resolution. *Points* and *whiskers* represent median and interquartile range, respectively. *p < 0.05 sham vs sepsis; (*)*p* = 0.05–0.06 sham vs sepsis. MCP = monocyte chemotactic protein, NGAL = neutrophil gelatinase-associated lipocalin.

Most proinflammatory cytokines were significantly elevated by 3 hours, including IL-1β, IL-6, MCP-1, and NGAL (**Fig. 3**). Apart from IL-6, all these cytokines remained elevated for at least 24 hours. The anti-inflammatory cytokine IL-10 was also significantly elevated at 3 hours. The earliest cytokine to peak was IL-1β at 3 hours, which remained elevated until 24 hours. Serum MCP-1 levels followed a similar pattern to IL-1β. IL-6, in contrast, peaked at 6 hours and fell sharply thereafter, to reach baseline levels by 48 hours. There was a trend toward an elevated IL-18 at 48 hours.

### Urine Biomarkers

**Figure 4.**
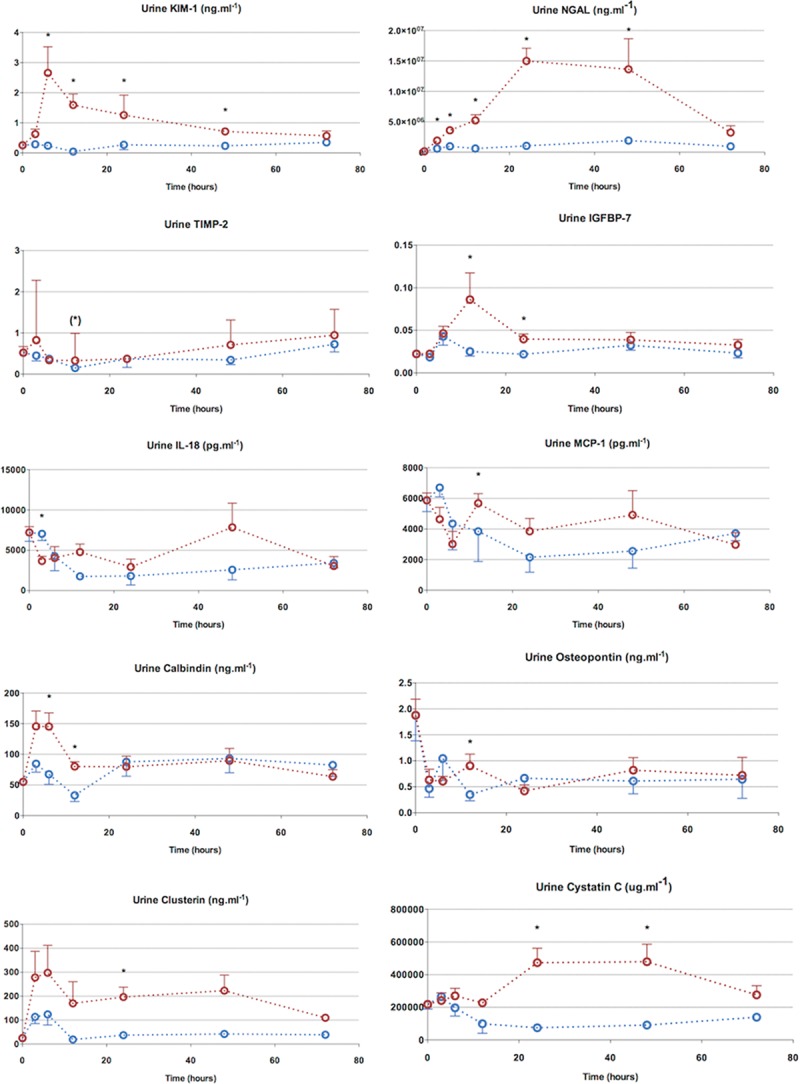
Seventy-two–hr characterization—urine biomarkers. Urine neutrophil gelatinase-associated lipocalin (NGAL) was the most sensitive marker, rising from 3 to 24 hr. Other biomarkers were also elevated before the rise in serum creatinine, though the magnitude of their rise was much less compared with NGAL. Urine interleukin (IL)-18 was not elevated in septic animals. *Points* and *whiskers* represent median and interquartile range, respectively. *p < 0.05 sham vs sepsis; (*)*p* = 0.05–0.06 sham vs sepsis. IGFPB-7 = insulin-like growth factor-binding protein 7, MCP = monocyte chemotactic protein.

Ten different urine biomarkers (KIM-1, NGAL, TIMP-2, IGFBP-7, IL-18, MCP-1, calbindin, clusterin, osteopontin, cystatin C) were measured in addition to serum biomarkers of renal function (serum urea, creatinine, and cystatin C) (**Fig. 4**). All urine biomarkers related to tubular cell injury, apart from TIMP-2, were significantly altered to varied degrees and with different kinetics. All biomarkers returned to levels approaching those observed in sham animals with clinical recovery. Apart from urine osteopontin and IL-18, all other urine biomarkers were elevated and at an earlier time point to serum creatinine (at 24 hr).

Urine NGAL was the earliest biomarker to rise (3 hr) with a sustained peak lasting from 24–48 hours. Urine KIM-1 and calbindin peaked at 6 hours, and fell thereafter, with calbindin reaching baseline values by 24 hours and KIM-1 by 72 hours. Although urine clusterin rose early (3 hr), it lacked discriminatory values due to variability in values. Urine cystatin C, a marker of glomerular filtration rate (GFR) and intact tubular reabsorption, was raised between 24 and 48 hours in septic animals.

Urine IGFBP-7, a marker of cell cycle arrest, was significantly elevated at 12 hours. This followed the rise seen in tubular injury markers but preceded the rise in functional markers of filtration (i.e., serum creatinine and cystatin C). TIMP-2, another cell cycle arrest marker, was also elevated at 12 hours, but only approached statistical significance. The fall in urinary levels of KIM-1 and calbindin at 24 hours seems to predict renal recovery. Although this time point coincided with a rise in serum creatinine, there was associated improvement in hemodynamics and a fall in proinflammatory cytokines (IL-1β, IL-6, IL-18).

In summary, some urine biomarkers including KIM-1 and calbindin rose early and then showed an early fall. On the other hand, NGAL also rose early, but peaked later, and remained elevated till clinical recovery. Cell cycle arrest markers rose after the injury markers and fell prior to clinical recovery. Urine cystatin C rose later, at the same time as serum creatinine (24 hours), suggestive of decreased renal functionality. Urine MCP-1, osteopontin, and IL-18 were elevated at various points in the clinical course but did not demonstrate any clear pattern of rise and fall.

As with current clinical use, it is unclear to what extent the urine biomarkers reflect what is filtered from the circulation into the urine, as opposed to de novo production within the kidney. There was no correlation between paired urine and serum levels of IL-18, MCP-1, or cystatin C. A modest positive correlation was seen between urine and serum NGAL values (*r*^2^ = 0.713; *p* < 0.001), even when including septic animals only (*r*^2^ = 0.654; *p* < 0.001).

### Renal Histology

**Figure 5.**
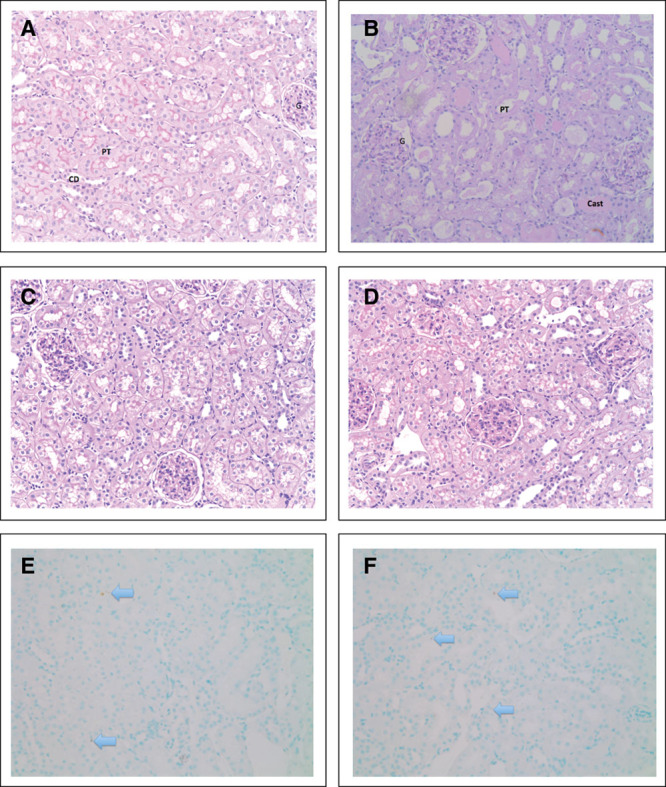
Histological assessment of rat kidneys (magnification ×20). **A**, Renal tissue without any significant damage. **B**, Renal tissue obtained from a hemorrhage-reperfusion model (Dyson et al [5]), showing several characteristics of acute tubular injury including dilated tubules with loss of brush border, ischemic glomeruli, and tubular casts. **C**, Kidney section from a 24-hr sham-operated rat. **D**, Kidney section from a 24-hr septic rat. **E**, Terminal deoxynucleotidyl transferase dUTP nick-end labeling (TUNEL) stain of naïve renal tissue shows two apoptotic cells per ×20 field. Apoptotic cells stain dark brown (*arrow*). **F**, TUNEL stain of 24-hr septic renal tissue shows occasional apoptotic cells. Apoptotic bodies seen mainly in proximal tubular epithelial cells (×20 magnification). Arrow = TUNEL positive cell, cast = tubular cast, CD = collecting duct, G = glomerulus, PT = proximal tubule.

The degree of injury at 24 hours was relatively mild (**Fig. 5**). Predominant findings included mild tubular dilatation and brush border loss. Tubular injury was patchy, among areas of normal histology. By comparison, histology is shown of a hemorrhage-reperfusion model performed in the laboratory in similarly aged male Wistar rats (Fig. 5B). TUNEL staining revealed minimal presence of cell death, with an average of two TUNEL positive cells per 20× magnification field. Where present, TUNEL positive cells were within proximal tubular epithelial cells (PTECs) (Fig. 5).

## DISCUSSION

Our study aim was to measure temporal changes in a panel of AKI biomarkers measured from the onset of sepsis through to recovery in a long-term fluid-resuscitated model of fecal peritonitis. This model demonstrates many hemodynamic, biochemical, and immunologic features consistent with clinical sepsis. As with human septic AKI, renal histology demonstrated minimal structural injury or cell death; influx of inflammatory cells was not seen. We report novel data showing an early change in markers of renal injury (urine NGAL, KIM-1, calbindin), followed by a marker of cell cycle arrest (urine IGFBP-7) and, finally, by functional markers of filtration (serum creatinine and cystatin C) (**Supplemental Fig. 1**, Supplemental Digital Content 1, http://links.lww.com/CCM/C456). Urine NGAL was the most sensitive marker among those studied, rising from 3 to 24 hours. The rise of the functional biomarker, serum cystatin C, at 12 hours implies a fall in GFR, whereas the concurrent fall in serum creatinine is suggestive of decreased creatinine production.

Serum urea and creatinine were initially elevated at 3 and 6 hours, and fell toward baseline values at 12 hours with IV fluid resuscitation. This is consistent with early hemoconcentration followed by a dilution effect. A correction factor can be applied to measured serum creatinine to correct for fluid administration (7) as acute hemodilution may mask the creatinine rise in early AKI (8). However, applying a correction factor for cumulative fluid balance over periods longer than a few hours lacks both a physiologic rationale and objective evidence of accuracy (9). After a large fluid bolus that increased circulating volume by 25%, 24-hour serum creatinine was only 2% below expected (9). The rise in serum cystatin C (consistent with a decrease in GFR) from 6 to 12 hours with concurrent falls in serum creatinine may be a consequence of decreased creatinine production (10). As with serum creatinine, sepsis decreases serum cystatin C production and increases nonrenal clearance (11). However, serum cystatin C had a faster rise and peaked more rapidly than creatinine. As such, serum cystatin C detects AKI early and better reflects inulin GFR in cecal ligation and puncture (CLP)-induced murine sepsis.

The temporal changes in cytokine levels in our model were similar to a cohort study of 1,886 subjects hospitalized with community-acquired pneumonia (12). There was an early peak of both pro- and anti-inflammatory cytokines followed by a decline in the proinflammatory cytokine profiles and persistence of the anti-inflammatory cytokine IL-10 though to recovery.

Food and Drug Administration–approved AKI biomarkers urine TIMP-2 and IGFBP-7 may be superior to NGAL in diagnosing AKI early in critically ill patients (13, 14). TIMP-2 and IGFBP-7 measured early in the setting of critical illness may also identify patients with AKI at increased risk of mortality or receipt of renal replacement therapy in the subsequent 9 months (15). In a CLP model of sepsis, the combination of TIMP-2 and IGFBP-7 has greater sensitivity in diagnosis of AKI compared with serum creatinine (6). However, the kinetics of these biomarkers in renal recovery has not been described.

Urine IL-18 is less promising than urine NGAL in septic AKI (13), and we report here similar findings. Two markers of distal tubular injury, calbindin and osteopontin, have not been previously evaluated in septic AKI. Urine calbindin was elevated at 6–12 hours, whereas urine osteopontin did not differ between septic and sham animals. Urine MCP-1 has also not been characterized in septic AKI; levels were significantly elevated between 12 and 18 hours.

A transient and modest, albeit statistically significant rise, was seen in urine osteopontin levels at 12 hours. Within the normal kidney, osteopontin is mainly present in the loop of Henlé and distal nephron (16), but in our model, proximal tubular injury predominated. In a renal ischemia-reperfusion injury model, the distribution of osteopontin in PTECs and distal TECs (DTECs) differed (17). While DTECs showed an early and persistent increase in osteopontin, the rise seen in PTECs was delayed and mostly associated with morphological regeneration. This suggests osteopontin may promote recovery via modulation of infiltrating cells and local responses by the PTEC. Ours, we believe, is the first study to measure urine osteopontin in septic AKI.

Consistent with other studies in both patients and animal models (18–20), the renal histology in our model shows disproportionately minimal parenchymal injury and apoptosis for the degree of functional impairment measured. There was no evidence of interstitial hypercellularity at any point to suggest significant immune cell infiltration.

Our preclinical model used a homogenous population of relatively young rats receiving an identical insult 24 hours after anesthesia and instrumentation. Patients with sepsis have a more variable genetic make-up, are usually older, with coexisting comorbid illnesses, and are receiving other nephrotoxic and renal physiology-modifying interventions. Other than hemodynamics and temperature, serial measurements were not made in the same animal as each time point represents the terminal experiment for collection of blood, renal tissue, and urine samples. We therefore cannot plot the trajectory of variables such as urine output or recovery from anuria over time. As the most unwell animals were anuric, urine biomarkers could not be measured in these animals.

Despite ample fluid resuscitation and becoming febrile, the rats in our peritonitis model do not develop a hyperdynamic circulation. We have previously shown in this model that significant myocardial depression yet maintained cardiac output (21). Many large animal models use an infusion of endotoxin or IV administration of live bacteria (rather than the more clinically representative fecal peritonitis insult), and these may produce a different circulatory/inflammatory profile.

We avoided antibiotic use in our study to avoid potential confounding from direct nephrotoxicity and renal impairment secondary to increased inflammation (22). We thus did not wish to confound the biomarker data in our study with a potential additional impact of antibiotics, which may add nephrotoxicity. Despite the nonuse of antibiotics, 80% of rats recovered with fluid resuscitation only. Similar data have been reported by Hollenberg et al (23) in a mouse CLP model. Of note, the outcome effectiveness of antibiotics is age-dependent (24).

Serum creatinine in the septic animals rose by only 50%, less than that seen clinically. However, in this fluid-resuscitated model, animals with a greater than 50% rise in serum creatinine at 24 hours tended to not survive much longer. In pilot studies, the creatinine rise seen at 24 hours could be reached by 6 hours in the absence of fluid resuscitation (data not shown). However, in these non-resuscitated animals, mortality rates were both very high and occurred much earlier, preventing study of the recovery phase.

Normalization to urinary creatinine concentration improved the prediction of developing AKI and outcome among critically ill patients, but provided no advantage in diagnosing established AKI (25). Similar findings were described in a rat model of drug-induced AKI (26). In this study, biomarker levels could not be corrected to urine creatinine concentration in many animals due to limited or absent urine output, particularly in the most severely affected.

We did not compare these biomarkers against other inflammatory, nonseptic insults, nor did we try to differentiate systemic from local production of the biomarker. Because of the need to terminally anesthetize the animals to obtain blood, urine, and tissue samples, we did not monitor biomarker levels in the same animal over time and thus could not assess the ability to prognosticate for the development of AKI.

No specific intervention currently exists for septic AKI. Intuitively, initiating therapies early would be of greater benefit, but this remains speculative. Time zero, the time of onset of AKI, is rarely known in patients. If the biomarker pattern we observed in our rat model holds true for patients, this may aid selection for interventions and potentially enhance the likelihood of therapeutic benefit. The temporal relationship of biomarker change described may shed some light into pathophysiologic mechanisms.

## Supplementary Material

## APPENDIX

### Fecal Slurry

The batch of slurry used for the entire set of experiments was identical. Fecal slurry was obtained from pooled stool from three healthy nonvegetarian donors. Immediately after collection, material was 1:1 diluted with a thioglycolate suspension and catalase for optimization of bacterial growth and inactivation of reactive oxygen species. Additionally, 10% glycerine was added and the suspension homogenized under anaerobic conditions. The slurry was then divided into aliquots and frozen at –80°C. Microbiology testing after thawing at different time intervals confirmed ongoing and similar bacterial growth of fecal flora. On the day of the experiment, aliquots were thawed, diluted with n-saline (1.2 mL slurry was diluted with 1.8 mL n-saline [2:3 dilution factor]), and 5 mL/kg body weight and injected intraperitoneally. The “potency” of fecal slurry does depend on the dilution factor. For this set of experiments, 1.2 mL slurry was diluted with 1.8 mL n-saline (2:3 dilution factor) and 5 mL/kg body weight was injected. A dose of 4.5–5.0 mL/kg was the LD50. The dose used in this set of experiments was 4 mL/kg, with a corresponding 20% mortality.
