# Supplementary figures and images for: Sequential Analysis of a Panel of Biomarkers and Pathologic Findings in a Resuscitated Rat Model of Sepsis and Recovery
Source: Crit Care Med. 2017 Jul 14;45(8):e821–30. doi: 10.1097/CCM.0000000000002381 (PMC5511729; doi:10.1097/CCM.0000000000002381)

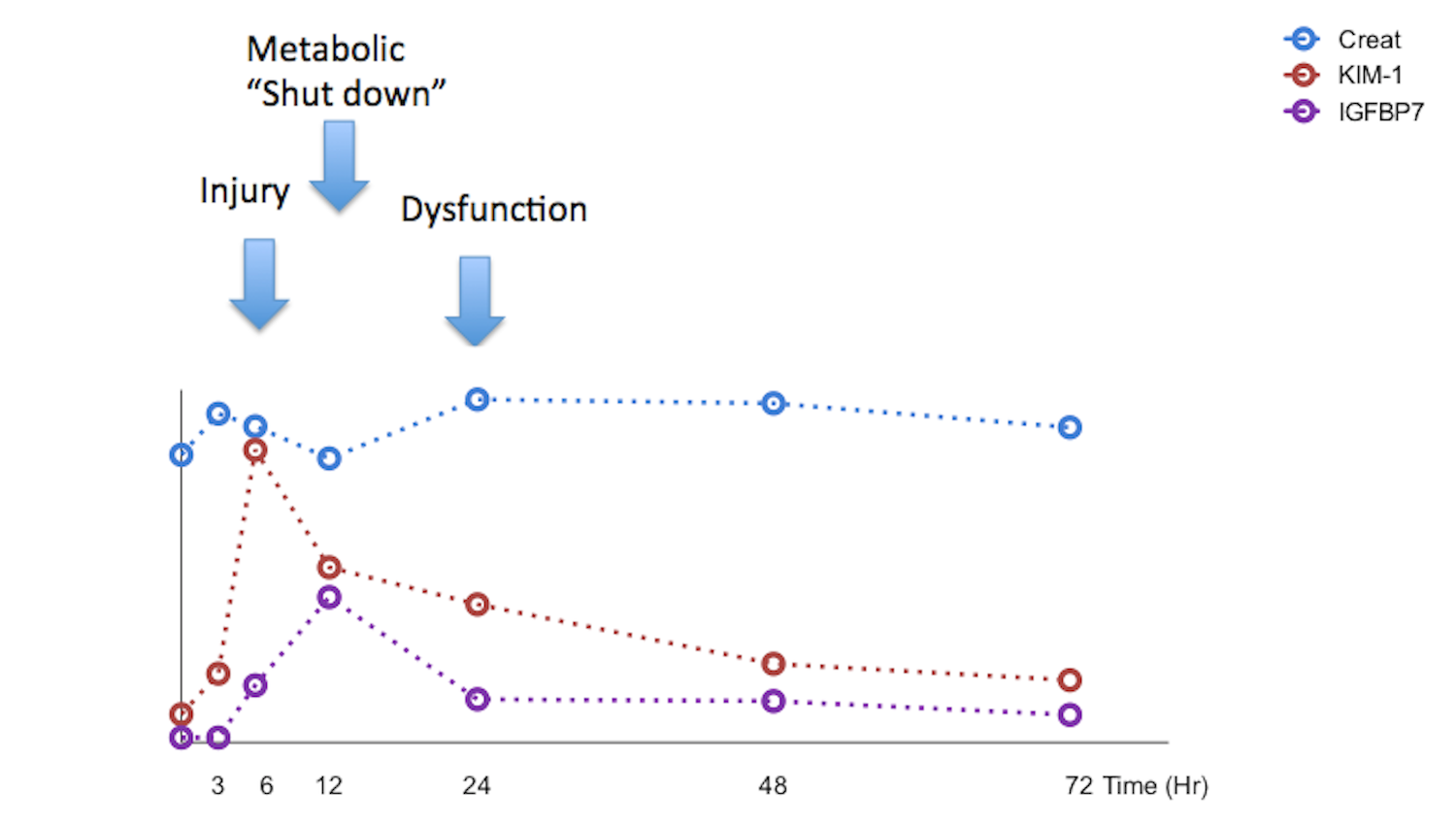

Supplement: Supplementary file 1 [file ccm-45-e821-s001.tiff]
